# *QuickStats:* Age-Adjusted Percentage* of Adults Aged ≥18 Years With Arthritis,^†^ by Sex and Race and Hispanic Origin — National Health Interview Survey,^§^ United States, 2021

**DOI:** 10.15585/mmwr.mm7204a6

**Published:** 2023-01-27

**Figure:**
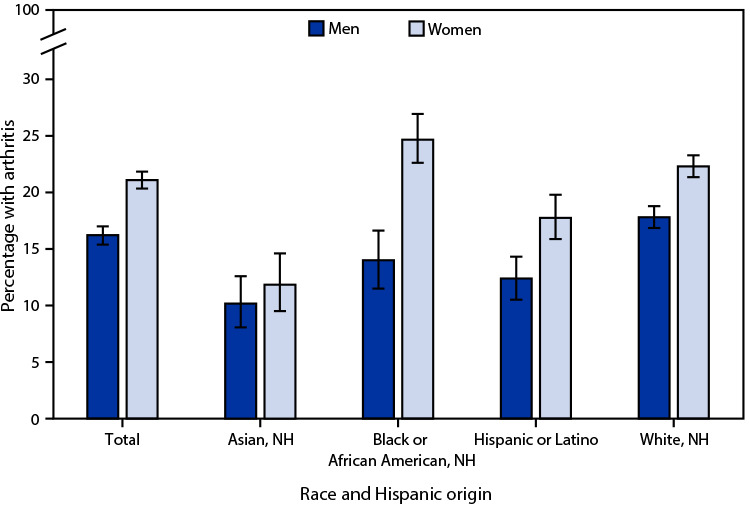
In 2021, among adults aged ≥18 years, women were more likely to have arthritis than were men (21.0% versus 16.2%). This pattern was consistent among non-Hispanic White (White) (22.2% versus 17.7%), non-Hispanic Black or African American (Black) (24.6% versus 13.9%), and Hispanic or Latino (17.7% versus 12.4%) adults. Among non-Hispanic Asian (Asian) adults, the higher rate of arthritis among women compared with men (11.8% versus 10.1%) was not statistically significant. Among women, Asian adults were least likely to have arthritis, whereas among men, Asian adults were less likely than White or Black adults to have arthritis.

**Source:** National Center for Health Statistics, National Health Interview Survey, 2021. https://www.cdc.gov/nchs/nhis/index.htm

